# Association of residential altitude with pure-tone hearing thresholds in plateau residents aged ≤50 years: a cross-sectional study

**DOI:** 10.3389/fneur.2026.1862718

**Published:** 2026-06-16

**Authors:** Qingping Zhang, Guoqiang Jia, Shanhong Li, Haiyan Liu, Yulin Ma, Wenqi Du, Juan Ye, Ying Zhang, Tana Wuren, Yi Wang, Bin Guo

**Affiliations:** 1 Department of Otolaryngology-Head and Neck Surgery, Affiliated Hospital of Qinghai University (The Clinical Medical School), Qinghai University, Xining, Qinghai, China; 2 Department of Health Management, Affiliated Hospital of Qinghai University (The Clinical Medical School), Qinghai University, Xining, Qinghai, China; 3 The Second People’s Hospital of Menyuan County, Menyuan County, Haibei Tibetan Autonomous Prefecture, Qinghai, China; 4 Second People’s Hospital of Haibei, Haibei Tibetan Autonomous Prefecture, Qinghai, China; 5 Medical College of Qinghai University, Xining, Qinghai, China; 6 Research Center for High Altitude Medicine, School of Medicine, Qinghai University, Xining, Qinghai, China; 7 Key Laboratory for Application of High-Altitude Medicine, Qinghai University, Xining, Qinghai, China; 8 Department of Otolaryngology, Peking Union Medical College Hospital, Chinese Academy of Medical Sciences and Peking Union Medical College, Beijing, China

**Keywords:** 8 kHz, cross-sectional study, hearing thresholds, high altitude, pure-tone audiometry, residential altitude

## Abstract

**Introduction:**

Residential altitude has been proposed as a potential determinant of auditory function, but evidence from long-term high-altitude residents remains limited.

**Methods:**

This cross-sectional study included 187 plateau residents aged ≤50 years undergoing health examination at the Affiliated Hospital of Qinghai University. Participants were classified into lower- (2,200–2,499 m, *n* = 79), middle- (2,500–3,499 m, *n* = 51), and higher-altitude (≥3,500 m, *n* = 57) strata according to long-term residential altitude. Mean bilateral thresholds at 250 Hz, 500 Hz, 1 kHz, 2 kHz, 4 kHz, and 8 kHz, together with LM-PTA and HF-PTA, were compared across groups. Complementary Kruskal–Wallis analyses, minimally and fully adjusted models, continuous-altitude analyses, exploratory quadratic-term analysis for PTA at 8 kHz, and repeated-measures ANCOVA were performed.

**Results:**

Unadjusted analyses showed more pronounced between-group differences at higher frequencies, with statistically significant differences for HF-PTA, PTA at 4 kHz, PTA at 8 kHz, and LM-PTA. Continuous-altitude models did not support a stable linear trend. In the fully adjusted model, altitude-stratified effects were no longer statistically significant for HF-PTA, PTA at 4 kHz, or LM-PTA, but remained statistically significant for PTA at 8 kHz (*p* = 0.032). Bonferroni-corrected comparisons showed that only the difference in PTA at 8 kHz between the lower- and middle-altitude strata remained statistically significant. The fully adjusted repeated-measures ANCOVA did not show a statistically significant Frequency × altitude group interaction.

**Discussion:**

Hearing-threshold differences across residential altitude strata were more apparent at higher frequencies in unadjusted analyses; after adjustment for age, sex, and other clinical factors, the most robust independent altitude-stratified association remained at 8 kHz.

## 1 Introduction

The defining physiological feature of the high-altitude environment is hypobaric hypoxia. As altitude increases, the decline in ambient oxygen partial pressure reduces arterial oxygen tension and oxygen saturation. This reduction may trigger maladaptive changes across the respiratory, circulatory, neurologic, and hematologic systems and contribute to clinical conditions such as acute mountain sickness, high-altitude cerebral edema, high-altitude pulmonary edema, and altitude-related hematologic alterations (1). More recent research in high-altitude medicine has further emphasized that the effects of hypobaric hypoxia are not confined to a single organ system, but instead involve multiple biological processes, including oxygen transport, microcirculatory regulation, metabolic reprogramming, and inflammation–oxidative stress pathways (2, 3). Against this background, the auditory system, as a metabolically active sensory system that is potentially sensitive to changes in oxygen supply, may also be affected by high-altitude exposure. However, compared with the cardiopulmonary, neurologic, and hematologic systems, clinical evidence linking high-altitude exposure to auditory alterations remains relatively limited.

Existing studies suggest that high-altitude exposure is indeed associated with changes in auditory function. A scoping review by Masè et al. (4) showed that most available studies reported elevated hearing thresholds, altered auditory evoked response latencies, or reduced otoacoustic emissions following high-altitude exposure; however, considerable heterogeneity in altitude level, exposure duration, study population, and testing modality has precluded a consistent conclusion as to whether high-altitude exposure primarily affects cochlear function or auditory brainstem conduction. Similarly, Yılmaz et al. (5) reported that chronic hypoxemia associated with chronic obstructive pulmonary disease (COPD) was associated with increased hearing thresholds, deterioration of DPOAEs, and prolongation of the ABR I–V interpeak interval, suggesting that reduced oxygen availability may affect both cochlear and brainstem auditory pathways. In human studies, Urbani and Lucertini (6) recorded auditory brainstem responses (ABRs) in six healthy volunteers before, during, and after 90 min of hypobaric chamber exposure simulating 5,184 m; notably, the principal significant finding was a shortening of the I–V interpeak interval during recovery relative to baseline, indicating that acute hypobaric hypoxia and the subsequent recovery phase may influence auditory brainstem conduction. Singh et al. (7) further reported that, compared with sea level, wave V absolute latency was prolonged at 3,200 m, whereas wave I and wave III latencies were also prolonged at 4,300 m, suggesting that high-altitude exposure may affect multiple components of the auditory afferent and brainstem pathways. Counter et al. (8) observed prolonged BAER absolute and interpeak latencies together with reduced wave I and wave V amplitudes in children residing long-term at high altitude, indicating possible changes in auditory neural synchrony and conduction physiology. At the cochlear level, Ide et al. (9) reported reductions of approximately 4 dB in both transient-evoked otoacoustic emissions (TEOAEs) and DPOAEs under hypobaric hypoxic conditions, supporting possible outer hair cell dysfunction. In addition, Fehrenbacher et al. (10) found aggravated temporary threshold shifts after noise exposure under high-altitude hypobaric hypoxic conditions, suggesting that the high-altitude environment may reduce inner-ear tolerance to additional injury.

Despite these observations, several important gaps remain. First, most previous studies have focused on short-term exposure, chamber simulations, or specific occupational or expedition populations, whereas data from real-world health examination populations living long-term at high altitude remain scarce. Second, although prior studies have used pure-tone audiometry, otoacoustic emissions, or auditory evoked responses separately, the relationship between residential altitude strata and pure-tone hearing-threshold profiles—particularly whether differences are more pronounced at higher frequencies—has not been systematically characterized. Third, age and sex are themselves major determinants of hearing thresholds. Population-based studies have consistently shown that hearing thresholds increase with age and that men generally have poorer high-frequency hearing than women, especially at 4 kHz and 8 kHz (11, 12). Therefore, any evaluation of the association between residential altitude and hearing thresholds in plateau populations requires adequate control for age, sex, and other clinical factors to minimize overestimation of the altitude effect. On this basis, the present study investigated the association between long-term residential altitude strata and pure-tone hearing-threshold characteristics in plateau residents aged 50 years or younger undergoing health examination, and further evaluated whether altitude strata remained independently associated with major audiological outcomes after adjustment for age, sex, and other clinical factors. Our aim was to provide clinical evidence for hearing monitoring and risk identification in high-altitude populations.

## 2 Materials and methods

### 2.1 Study design and participants

This was a single-center cross-sectional study. Participants were recruited from individuals undergoing routine health examination at the Health Examination Center of the Affiliated Hospital of Qinghai University. The study was approved by the Ethics Committee of the Affiliated Hospital of Qinghai University (The Clinical Medical School) (approval no. P-SL-2024-013), and written informed consent was obtained from all participants. Eligible participants were aged 50 years or younger, resided in the Qinghai Plateau region, and had completed pure-tone audiometry. All participants underwent otoscopic examination and tympanometric screening, and only those with type A tympanograms were included to minimize the influence of conductive hearing abnormalities. The exclusion criteria were as follows: (1) otologic diseases or histories that could affect hearing evaluation, including outer or middle ear disease, a definite history of sensorineural tinnitus or hearing loss, Ménière’s disease, mumps, and Ramsay Hunt syndrome; (2) a definite history of occupational noise exposure or ototoxic drug exposure; (3) nasal diseases that might affect hearing via Eustachian tube dysfunction, such as sinusitis or allergic rhinitis; (4) other neurologic or systemic conditions that might substantially influence hearing-threshold assessment, including cerebrovascular events, traumatic brain injury, cerebellopontine angle tumors, nasopharyngeal carcinoma, diabetes mellitus, and hepatic or renal dysfunction. A total of 187 eligible participants were finally included.

### 2.2 Clinical data collection

Demographic and clinical data were collected, including age, sex, ethnicity, educational level, smoking history, alcohol consumption, hypertension, hyperlipidemia, and obstructive sleep apnea-hypopnea syndrome (OSAHS). Long-term residential altitude and duration of residence were also recorded. Among participants who underwent blood testing, hematologic indices were additionally collected, including hemoglobin (HGB), red blood cell count (RBC), and hematocrit (HCT).

### 2.3 Pure-tone audiometry

Pure-tone audiometry was performed using a calibrated diagnostic audiometer in accordance with the GB7341 national standard. All examinations were conducted in a soundproof booth with ambient background noise controlled below 25 dB. Before testing, a trained audiologist explained the procedure and response instructions to each participant. Participants were required to avoid noise exposure for at least 24 h before the examination. Air-conduction thresholds were measured first, followed by bone-conduction thresholds when clinically indicated. Air-conduction thresholds were determined using a down-10/up-5 bracketing procedure. The initial stimulus intensity at each frequency was 40 dB HL. If the participant detected the stimulus, intensity was decreased in 10-dB steps until no response was obtained and then increased in 5-dB steps to identify the lowest reproducible response level. After threshold identification, the intensity was decreased by 5 dB to confirm no response and then returned to the threshold level for repeat verification. The testing sequence started at 1,000 Hz, followed by 2,000 Hz, 4,000 Hz, 8,000 Hz, 500 Hz, and 250 Hz, with 1,000 Hz retested at the end to verify response reliability. This sequence followed the routine clinical protocol of our audiology unit, with 1,000 Hz used first to familiarize participants with the task.

### 2.4 Audiological assessment and outcome definitions

To characterize hearing status across different frequency ranges, the following audiological outcomes were prespecified: PTA at 250 Hz, PTA at 500 Hz, PTA at 1 kHz, PTA at 2 kHz, PTA at 4 kHz, and PTA at 8 kHz represented the mean bilateral hearing threshold at each corresponding frequency.

LM-PTA was defined as:

LM-PTA = (left 500 Hz + left 1,000 Hz + left 2,000 Hz + right 500 Hz + right 1,000 Hz + right 2,000 Hz) / 6.

HF-PTA was defined as:

HF-PTA = (left 4 kHz + left 8 kHz + right 4 kHz + right 8 kHz) / 4.

### 2.5 Altitude exposure stratification

According to commonly used altitude classification frameworks in high-altitude medicine, and taking into account that all participants were long-term residents of the Qinghai Plateau with a minimum residential altitude of approximately 2,200 m, residential altitude was categorized into three exposure strata: 2,200–2,499 m, 2,500–3,499 m, and ≥3,500 m. Altitude strata were used as the primary exposure representation for descriptive comparisons and adjusted between-group analyses.

In addition, residential altitude was modeled as a continuous variable (per 1,000 m) in supplementary analyses to assess linear trends. For the key outcome PTA at 8 kHz, centered residential altitude and its quadratic term were further incorporated to explore potential nonlinearity (Supplementary Table S1). Restricted cubic spline (RCS) analysis was also performed as a supplementary nonlinear approach to visualize possible non-linear associations between residential altitude and PTA at 8 kHz (Supplementary Figure S1).

### 2.6 Statistical analysis

The main statistical analyses were performed using SPSS version 27.0, and the restricted cubic spline analysis for PTA at 8 kHz was conducted using R. Continuous variables were summarized as mean ± standard deviation or median (Q1, Q3), depending on their distribution, whereas categorical variables were summarized as counts and percentages. For comparisons of baseline characteristics among the three altitude strata, continuous variables were analyzed using one-way analysis of variance or nonparametric tests as appropriate, and categorical variables were compared using the chi-square test. In unadjusted comparisons of audiological outcomes, one-way analysis of variance followed by Bonferroni *post hoc* correction was used when homogeneity of variance was satisfied; otherwise, Welch’s analysis of variance followed by Games-Howell *post hoc* testing was applied. Kruskal–Wallis tests were additionally performed as complementary nonparametric analyses to examine whether the unadjusted group comparisons were consistent under a nonparametric approach. For the major audiological outcomes (HF-PTA, PTA at 4 kHz, PTA at 8 kHz, and LM-PTA), general linear models (GLMs) were constructed using residential altitude in both categorical form (2,200–2,499 m, 2,500–3,499 m, and ≥3,500 m) and continuous form (per 1,000 m). Altitude-stratified models were used for the primary between-group comparisons, including a minimally adjusted model and a fully adjusted model. The minimally adjusted model included altitude strata, age, and sex. The fully adjusted model additionally included ethnicity, smoking history, alcohol consumption, hypertension, hyperlipidemia, OSAHS, and duration of residence. Continuous-altitude models were used to assess linear trends. Model results are presented as overall *p* values, estimated marginal means, and Bonferroni-corrected pairwise comparisons. For PTA at 8 kHz, centered residential altitude and its quadratic term were further entered into the model as an exploratory analysis of potential nonlinearity, and parameter estimates, 95% confidence intervals, and *p* values were reported. RCS analysis was used as a supplementary approach to evaluate and visualize the possibility of a non-linear dose–response relationship between residential altitude and PTA at 8 kHz. To further examine whether altitude-related hearing-threshold differences showed an overall frequency-specific pattern, repeated-measures analysis of covariance (ANCOVA) was performed as a sensitivity analysis using PTA at 250 Hz, PTA at 500 Hz, PTA at 1 kHz, PTA at 2 kHz, PTA at 4 kHz, and PTA at 8 kHz as within-subject repeated outcomes. In the fully adjusted model, age and duration of residence were entered as covariates, whereas altitude strata, sex, ethnicity, smoking history, alcohol consumption, hypertension, hyperlipidemia, and OSAHS were entered as between-subject factors. If Mauchly’s test indicated violation of the sphericity assumption, Greenhouse–Geisser-corrected results were reported.

All tests were two-sided, and a *p* value < 0.05 was considered statistically significant. Regression analyses were conducted using complete-case analysis.

## 3 Results

### 3.1 Baseline characteristics of the study population

A total of 187 plateau residents aged 50 years or younger undergoing health examination were ultimately included in this study. According to long-term residential altitude, participants were classified into a lower-altitude stratum (2,200–2,499 m, *n* = 79), a middle-altitude stratum (2,500–3,499 m, *n* = 51), and a higher-altitude stratum (≥3,500 m, *n* = 57). The mean ages of the three groups were 31.38 ± 7.02, 35.06 ± 8.92, and 35.96 ± 9.09 years, respectively, with a statistically significant between-group difference (*p* = 0.010). The mean durations of residence were 23.06 ± 11.01, 23.14 ± 13.66, and 25.84 ± 14.31 years, respectively, with no statistically significant between-group difference (*p* = 0.130). The numbers of participants who underwent blood testing were 66, 49, and 49 in the lower-, middle-, and higher-altitude strata, respectively. Median HGB levels were 169.5 (154.0, 185.0) g/L, 175.0 (165.0, 189.5) g/L, and 165.0 (150.0, 180.5) g/L; median RBC counts were 5.41 (4.91, 5.74) × 10^12^/L, 5.49 (5.17, 5.92) × 10^12^/L, and 5.35 (4.95, 5.66) × 10^12^/L; and median HCT values were 49.20% (44.60, 51.97), 50.70% (47.18, 53.03), and 48.00% (44.33, 52.28), respectively. Overall, erythrocyte-related indices did not show a monotonic increase across the three altitude strata. Among these indices, HCT differed significantly among groups (*p* = 0.025), whereas HGB and RBC showed only borderline or non-significant differences. In addition, the ethnic composition differed significantly across the three groups, with Han participants predominating in the lower- and middle-altitude strata and Tibetan participants predominating in the higher-altitude stratum (*p* < 0.001) (Table 1).

**Table 1:** Baseline characteristics of participants across residential altitude strata.

Variable	Overall (*n* = 187)	Lower-altitude stratum (*n* = 79)	Middle-altitude stratum (*n* = 51)	Higher-altitude stratum (*n* = 57)	*p* value
Age, years	33.85 ± 8.46	31.38 ± 7.02	35.06 ± 8.92	35.96 ± 9.09	0.010
Duration of residence, years	23.99 ± 12.76	23.06 ± 11.01	23.14 ± 13.66	25.84 ± 14.31	0.130
Male, *n* (%)	123 (65.8)	47 (59.5)	39 (76.5)	37 (64.9)	0.136
Female, *n* (%)	64 (34.2)	32 (40.5)	12 (23.5)	20 (35.1)	
Ethnicity, *n* (%)					<0.001
Han	112 (59.9)	62 (78.5)	38 (74.5)	12 (21.1)	
Tibetan	57 (30.5)	5 (6.3)	8 (15.7)	44 (77.2)	
Other ethnic groups	18 (9.6)	12 (15.2)	5 (9.8)	1 (1.8)	
Smoking history, *n* (%)					0.072
No	125 (66.8)	54 (68.4)	28 (54.9)	43 (75.4)	
Yes	62 (33.2)	25 (31.6)	23 (45.1)	14 (24.6)	
Alcohol consumption, *n* (%)					0.199
No	173 (92.5)	70 (88.6)	48 (94.1)	55 (96.5)	
Yes	14 (7.5)	9 (11.4)	3 (5.9)	2 (3.5)	
Hypertension, *n* (%)					0.630
No	173 (92.5)	73 (92.4)	46 (90.2)	54 (94.7)	
Yes	13 (7.0)	5 (6.3)	5 (9.8)	3 (5.3)	
Hyperlipidemia, *n* (%)					0.507
No	155 (82.9)	64 (81.0)	41 (80.4)	50 (87.7)	
Yes	32 (17.1)	15 (19.0)	10 (19.6)	7 (12.3)	
OSAHS, *n* (%)					0.197
No	180 (96.3)	75 (94.9)	48 (94.1)	57 (100.0)	
Yes	7 (3.7)	4 (5.1)	3 (5.9)	0 (0.0)	
HGB, g/L	170.0 (150.0, 181.0)	169.5 (154.0, 185.0)	175.0 (165.0, 189.5)	165.0 (150.0, 180.5)	0.052
RBC, ×10^12^/L	5.41 (4.96, 5.75)	5.41 (4.91, 5.74)	5.49 (5.17, 5.92)	5.35 (4.95, 5.66)	0.087
HCT, %	49.25 (44.83, 52.40)	49.20 (44.60, 51.97)	50.70 (47.18, 53.03)	48.00 (44.33, 52.28)	0.025

Continuous variables are presented as mean ± standard deviation or median (Q1, Q3), as appropriate according to distribution. Categorical variables are presented as *n* (%). Hematologic indices were analyzed only among participants who underwent blood testing. Some variables contained missing values; percentages were calculated based on available data.

### 3.2 Unadjusted comparisons of hearing thresholds across residential altitude strata

**Figure 1:**
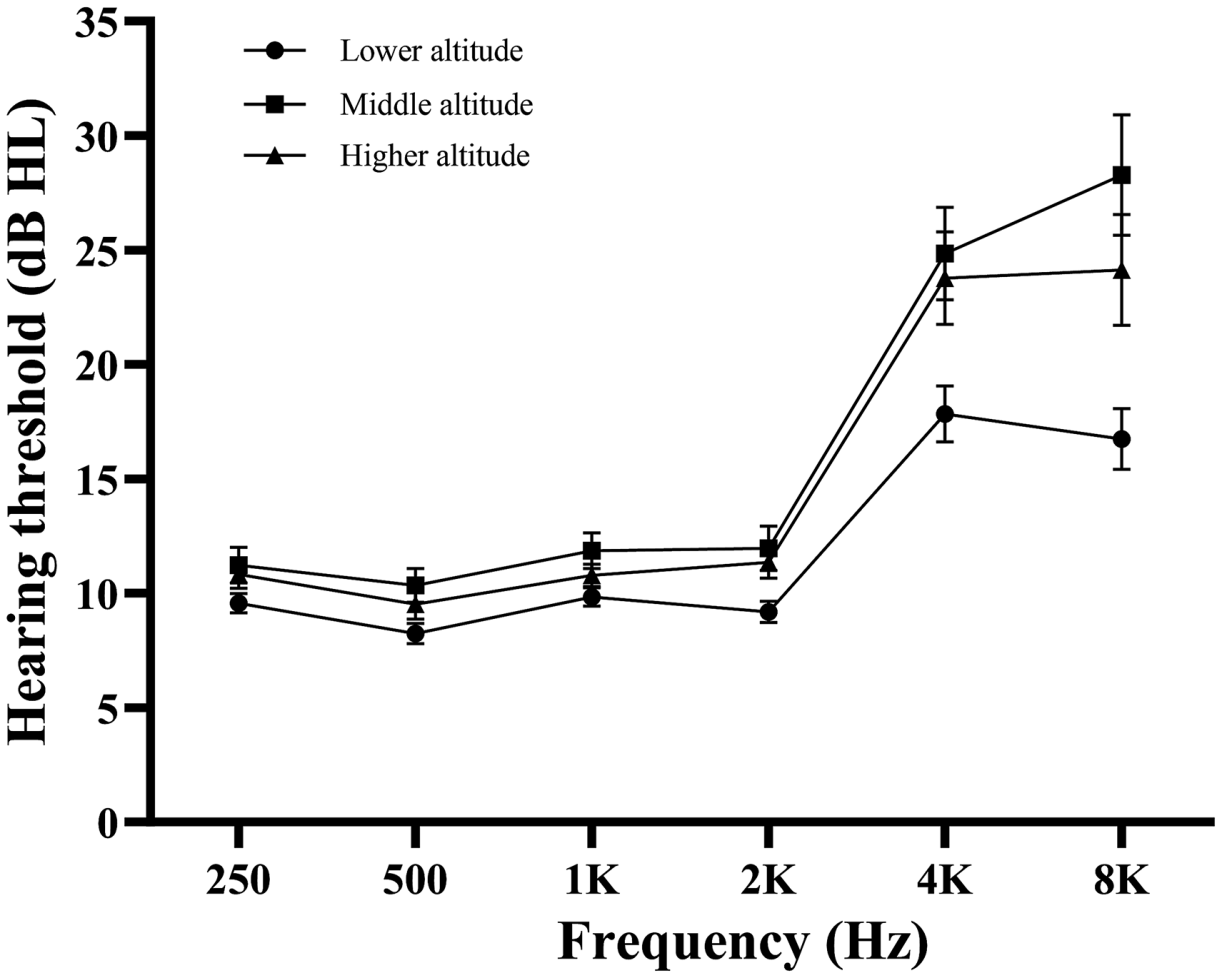
Mean bilateral pure-tone hearing-threshold curves across residential altitude strata. The x-axis indicates frequency (Hz), and the y-axis indicates mean bilateral hearing threshold (dB HL). Data are presented as mean ± SEM. The lower-, middle-, and higher-altitude strata are indicated by different point symbols.

Unadjusted analyses showed that hearing thresholds differed across residential altitude strata, with more pronounced differences at higher frequencies. Significant between-group differences were observed for HF-PTA (*p* = 0.001), PTA at 4 kHz (*p* = 0.024), PTA at 8 kHz (*p* < 0.001), and LM-PTA (*p* = 0.007). *Post hoc* pairwise comparisons showed that both HF-PTA and PTA at 8 kHz were significantly lower in the lower-altitude stratum than in the middle- and higher-altitude strata, whereas the between-group differences in PTA at 4 kHz and LM-PTA were relatively weaker. Analyses of individual low- and mid-frequency outcomes showed no clear between-group difference at 250 Hz. At 500 Hz and 2 kHz, some differences were observed in the unadjusted parametric comparisons, with the signal at 2 kHz being relatively more evident; however, these findings were overall less robust than those at higher frequencies (Table 2; Figure 1).

**Table 2:** Unadjusted audiological comparisons across residential altitude strata.

Outcome (dB HL)	Lower-altitude stratum (2,200–2,499 m, *n* = 79)	Middle-altitude stratum (2,500–3,499 m, *n* = 51)	Higher-altitude stratum (≥3,500 m, *n* = 57)	Overall *P* value	Kruskal-Wallis *P* value	Significant pairwise comparisons
PTA at 250 Hz	9.56 ± 3.81	11.23 ± 5.55	10.83 ± 4.61	0.086	0.196	None
PTA at 500 Hz	8.23 ± 4.05	10.34 ± 5.39	9.52 ± 5.01	0.040	0.105	Lower vs. middle
PTA at 1 kHz	9.84 ± 3.54	11.86 ± 5.59	10.79 ± 3.63	0.053	0.127	None
PTA at 2 kHz	9.18 ± 4.15	11.96 ± 6.92	11.36 ± 5.20	0.006	0.051	Lower vs. middle; lower vs. higher
LM-PTA	9.08 ± 3.27	11.39 ± 5.43	10.56 ± 3.68	0.007	0.036	Lower vs. middle; lower vs. higher
PTA at 4 kHz	18.58 ± 12.54	24.71 ± 14.18	23.95 ± 16.04	0.024	0.008	Lower vs. middle
PTA at 8 kHz	17.25 ± 12.13	28.09 ± 18.48	23.99 ± 17.53	<0.001	<0.001	Lower vs. middle; lower vs. higher
HF-PTA	17.91 ± 11.02	26.40 ± 14.93	23.97 ± 15.77	0.001	<0.001	Lower vs. middle; lower vs. higher

Data are presented as mean ± standard deviation. For the overall *P* values, one-way ANOVA with Bonferroni *post hoc* correction was used when homogeneity of variance was satisfied, whereas Welch’s ANOVA with Games-Howell *post hoc* testing was used when homogeneity of variance was violated. Kruskal–Wallis testing was performed as a complementary nonparametric analysis. The findings at 500 Hz and 2 kHz were relatively less robust and should therefore be interpreted with caution: the 500-Hz result was statistically significant in the parametric analysis but was not supported by the complementary Kruskal–Wallis analysis, whereas the 2-kHz result was statistically significant in the parametric analysis but showed only borderline significance in the Kruskal-Wallis test (*p* = 0.051). HF-PTA was defined as (left 4 kHz+ left 8 kHz+ right 4 kHz+ right 8 kHz)/4; LM-PTA was defined as (left 500 Hz+ left 1,000 Hz+ left 2,000 Hz+ right 500 Hz+ right 1,000 Hz+ right 2,000 Hz)/6.

### 3.3 Complementary nonparametric analysis: Kruskal–Wallis test

The complementary nonparametric analyses using the Kruskal–Wallis test were broadly consistent with the primary parametric comparisons. Between-group differences remained statistically significant for HF-PTA, PTA at 4 kHz, PTA at 8 kHz, and LM-PTA in the nonparametric analyses, whereas the findings at 500 Hz and 2 kHz were relatively less robust, with the result at 2 kHz showing only borderline statistical significance. Overall, these results suggest that altitude-related hearing-threshold differences were most stable at higher frequencies, may extend to some mid frequencies, and were not clearly evident at the lowest frequency tested.

### 3.4 Adjusted associations between residential altitude strata and major audiological outcomes

**Figure 2:**
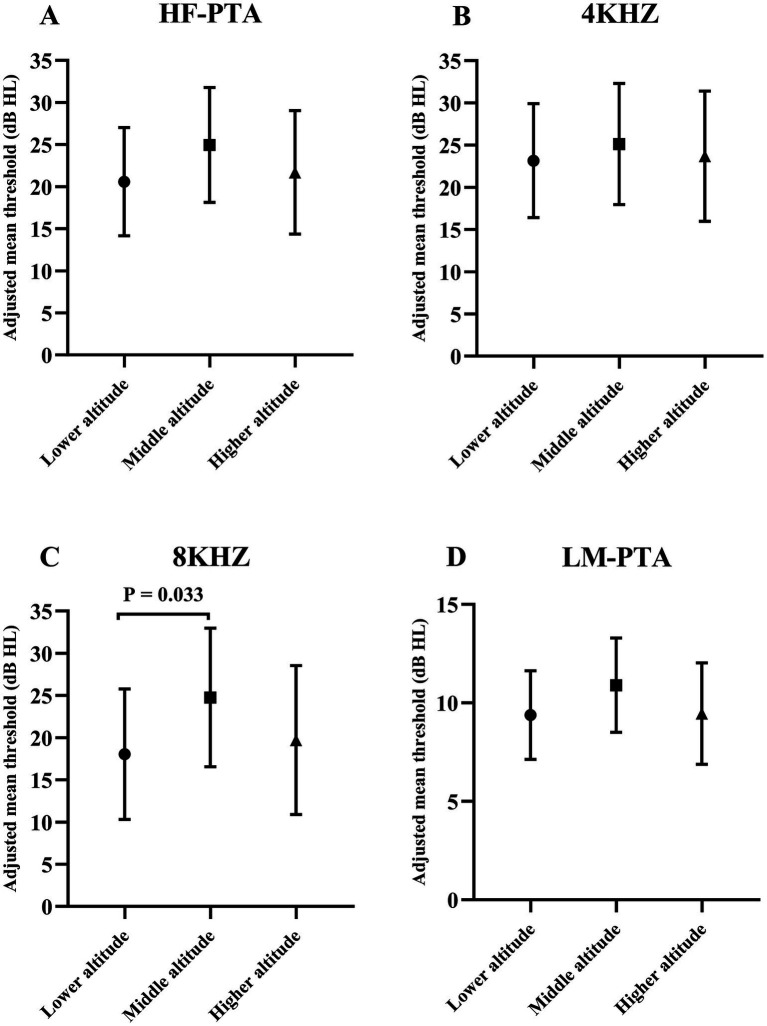
Adjusted marginal means of major audiological outcomes across residential altitude strata. **(A)** HF-PTA; **(B)** PTA at 4 kHz; **(C)** PTA at 8 kHz; and **(D)** LM-PTA. Points indicate estimated marginal means from Model 3, and error bars indicate 95% confidence intervals. Only PTA at 8 kHz retained an independent altitude-stratified effect in the fully adjusted model, with a significant difference between the lower- and middle-altitude strata.

When residential altitude was entered as a continuous variable in fully adjusted general linear models, no stable linear trend was observed between altitude and the major audiological outcomes, including HF-PTA, PTA at 4 kHz, PTA at 8 kHz, and LM-PTA. For example, the regression coefficient for PTA at 8 kHz was approximately 2.43 dB per 1,000 m, but this did not reach statistical significance (Supplementary Table S1). Therefore, subsequent adjusted analyses focused on altitude-stratified models. In the minimally adjusted model (altitude strata + age + sex), the altitude-stratified effect was only borderline for HF-PTA (*p* = 0.066), was not statistically significant for PTA at 4 kHz (*p* = 0.394), remained significant for LM-PTA (*p* = 0.022), and remained statistically significant for PTA at 8 kHz (*p* = 0.021). In the fully adjusted model (further including ethnicity, smoking history, alcohol consumption, hypertension, hyperlipidemia, OSAHS, and duration of residence), the altitude-stratified effects were no longer statistically significant for HF-PTA (*p* = 0.123), PTA at 4 kHz (*p* = 0.673), or LM-PTA (*p* = 0.099), whereas PTA at 8 kHz retained an independent association with altitude strata (*p* = 0.032). Bonferroni-corrected pairwise comparisons showed that only the difference in PTA at 8 kHz between the lower- and middle-altitude strata remained statistically significant, whereas no other pairwise contrasts reached significance (Table 3). The exploratory quadratic-term analysis for PTA at 8 kHz showed borderline significance for both the centered linear altitude term (β = 4.50, *p* = 0.059) and the quadratic term (β = −5.64, *p* = 0.061), suggesting that the relationship between residential altitude and PTA at 8 kHz may not be simply linear and may instead be non-monotonic (Supplementary Figure S1; Supplementary Table S1). In the GLM and repeated-measures ANCOVA models, age and sex were consistently associated with multiple audiological outcomes, and men had higher thresholds than women for HF-PTA, PTA at 4 kHz, and PTA at 8 kHz (Table 3; Figure 2).

**Table 3:** Associations between residential altitude strata and major audiological outcomes: unadjusted, minimally adjusted, and fully adjusted models.

Outcome	Model 1: Overall *P* value	Model 2: Overall *P* value	Model 2: Adjusted means (lower/middle/higher)	Model 2: Significant pairwise comparisons	Model 3: Overall *P* value	Model 3: Adjusted means (lower/middle/higher)	Model 3: Significant pairwise comparisons
HF-PTA	0.001	0.066	18.31/23.18/21.18	None	0.123	20.59/24.94/21.70	None
PTA at 4 kHz	0.024	0.394	18.74/21.36/21.21	None	0.673	23.15/25.13/23.68	None
PTA at 8 kHz	<0.001	0.021	17.88/24.99/21.15	Lower vs. middle	0.032	18.03/24.76/19.71	Lower vs. middle
LM-PTA	0.007	0.022	9.38/11.40/10.38	Lower vs. middle	0.099	9.38/10.90/9.46	None

Model 1 represents unadjusted between-group comparisons using one-way ANOVA or Welch’s ANOVA, depending on homogeneity of variance. Model 2 is the minimally adjusted model including altitude strata, age, and sex. Model 3 is the fully adjusted model including altitude strata, age, sex, ethnicity, smoking history, alcohol consumption, hypertension, hyperlipidemia, OSAHS, and duration of residence. Adjusted means are estimated marginal means. Pairwise comparisons were Bonferroni-corrected.

To further examine whether altitude-related hearing-threshold differences showed an overall frequency-specific pattern, repeated-measures ANCOVA was performed as a sensitivity analysis using PTA at 250 Hz, PTA at 500 Hz, PTA at 1 kHz, PTA at 2 kHz, PTA at 4 kHz, and PTA at 8 kHz as within-subject repeated outcomes. In the fully adjusted model, age and duration of residence were included as covariates, and altitude strata, sex, ethnicity, smoking history, alcohol consumption, hypertension, hyperlipidemia, and OSAHS were included as between-subject factors. Mauchly’s test indicated violation of the sphericity assumption (W = 0.0116, χ^2^ = 762.20, df = 14, *p* < 0.001); therefore, Greenhouse–Geisser-corrected results were reported. After correction, the Frequency × altitude group interaction remained non-significant [*F*(4.137, 357.830) = 1.273, *p* = 0.280, partial η^2^ = 0.015], indicating that the hearing-threshold differences across residential altitude strata did not show a clear overall frequency-specific interaction pattern (Supplementary Table S2). In contrast, both the Frequency × age and Frequency × sex interactions were statistically significant (*p* = 0.001 and *p* < 0.001, respectively), whereas the interactions of Frequency with duration of residence, ethnicity, smoking history, alcohol consumption, hypertension, hyperlipidemia, and OSAHS were all non-significant. Taken together, the repeated-measures analysis supports the interpretation that the effects of age and sex on hearing thresholds vary across frequencies, whereas no statistically significant overall interaction was observed between residential altitude strata and frequency. Importantly, the non-significant Frequency × altitude group interaction does not negate the independent altitude-stratified effect observed for PTA at 8 kHz in the single-outcome GLMs; rather, it indicates that the overall frequency profile did not differ significantly across altitude strata after multivariable adjustment.

## 4 Discussion

The main finding of this study is that, among plateau residents aged 50 years or younger undergoing health examination, pure-tone hearing thresholds differed across long-term residential altitude strata, and these differences were more pronounced at higher frequencies in the unadjusted analyses. The complementary Kruskal–Wallis analyses showed a generally consistent pattern for HF-PTA, PTA at 4 kHz, PTA at 8 kHz, and LM-PTA, supporting the robustness of the higher-frequency signal. More importantly, after adjustment for age, sex, and other clinical factors, the altitude-stratified effect was retained mainly at 8 kHz, whereas the between-group differences in HF-PTA, PTA at 4 kHz, and LM-PTA were markedly attenuated or no longer statistically significant. In addition, continuous-altitude models did not support a stable linear dose–response relationship, and both the quadratic-term analysis and spline analysis for PTA at 8 kHz suggested only exploratory evidence of nonlinearity. Taken together, these findings indicate that the association between residential altitude and hearing thresholds may not be adequately captured by a simple linear term and may instead be better characterized as a stratified or non-monotonic pattern, with 8 kHz representing the most stable associated phenotype. Furthermore, the fully adjusted repeated-measures ANCOVA did not show a statistically significant Frequency × altitude group interaction, indicating that this study does not support a clear overall frequency-specific interaction pattern (Supplementary Table S2). Accordingly, the more cautious interpretation is that altitude-related hearing-threshold differences were more prominent at higher frequencies in descriptive and single-outcome models, and that after adjustment for confounders, the most consistent association was observed at 8 kHz. Therefore, the present findings should be interpreted primarily as an adjusted association between residential altitude strata and PTA at 8 kHz, rather than as definitive evidence of a broad altitude-related frequency-specific hearing-loss pattern.

These findings are broadly consistent with previous studies on the effects of high-altitude exposure on auditory function, while also extending the literature by providing data from real-world long-term high-altitude residents. The scoping review by Masè et al. (4), which included 17 studies and 888 participants, indicated that most studies observed elevated hearing thresholds, altered auditory evoked response latencies, or reduced otoacoustic emissions after high-altitude exposure. Studies by Urbani and Lucertini (6) and Singh et al. (7) suggested that hypobaric hypoxia may affect auditory brainstem conduction parameters, whereas Ide et al. (9) provided evidence from otoacoustic emissions supporting possible cochlear involvement. Compared with these studies, which mainly focused on acute exposure, hypobaric chamber experiments, or special populations, the value of the present study lies in showing, in a real-world health examination population aged ≤50 years living long-term at high altitude, that altitude-related hearing-threshold differences were more evident at higher frequencies in unadjusted analyses, whereas after multivariable adjustment, the signal was retained mainly at 8 kHz, a more clinically interpretable audiological outcome.

The persistence of an altitude-stratified effect at 8 kHz in the fully adjusted model, together with the attenuation of the effects for 4 kHz, HF-PTA, and LM-PTA, is biologically plausible. The cochlear base is responsible for high-frequency sound encoding, and outer hair cells in the basal turn are generally considered more vulnerable than those in the apical turn across multiple injury models. Fettiplace and Nam (13) suggested that high-frequency outer hair cells in the basal turn are smaller and operate with greater mechanoelectrical transduction currents, making them more susceptible to imbalance in calcium homeostasis and metabolic load, and therefore more vulnerable to noise, ototoxic agents, and other forms of stress. In parallel, Shi’s review (14) of cochlear microcirculation emphasized that the microcirculation of the cochlear lateral wall and stria vascularis is essential for maintaining the endocochlear potential and cochlear homeostasis, and that hypoperfusion, blood-labyrinth barrier disruption, or lateral-wall dysfunction may preferentially impair metabolically demanding cochlear regions. In studies of age-related hearing loss, Lang et al. (15) further suggested that the stria vascularis is an early site of degeneration and immune dysregulation, which may contribute to threshold elevation through disruption of lateral-wall homeostasis. Although these data derive primarily from presbycusis, noise-induced hearing loss, and other cochlear injury models rather than directly from long-term high-altitude exposure, they provide a biologically plausible framework for understanding why 8 kHz may be vulnerable in the present study. These mechanistic interpretations remain speculative because objective cochlear or auditory pathway measures, such as OAE and ABR, were not available in the present study.

One noteworthy finding of the present study is that hearing thresholds in the higher-altitude stratum did not show progressive worsening; rather, for some outcomes they were lower than those observed in the middle-altitude stratum. This pattern suggests that the relationship between residential altitude and hearing thresholds may not follow a simple linear dose–response gradient, but may instead be non-monotonic. One possible explanation is ethnicity-related high-altitude adaptation, as the proportion of Tibetan participants was substantially higher in the higher-altitude stratum, and Tibetan adaptation to high-altitude environments has been associated with a distinct hypoxia–hemoglobin regulatory phenotype. The non-monotonic pattern of hematologic indices across altitude strata may also be relevant to altitude-related physiological adaptation; however, this interpretation remains exploratory because hematologic data were not available for all participants, no formal mediation analysis was performed, and hematologic indices did not demonstrate stable independent explanatory effects in the multivariable models. Recent studies in high-altitude physiology have similarly emphasized that hemoglobin responses to high-altitude exposure are heterogeneous and influenced by multiple factors, including plasma-volume changes, exposure duration, individual adaptive status, and genetic background, and therefore should not be regarded as a single, persistent, and linear indicator of adaptation (3, 16). Therefore, these hematologic findings should be regarded as hypothesis-generating rather than mechanistic evidence.

In the GLM and repeated-measures ANCOVA models, age and sex were consistently associated with multiple audiological outcomes, which is consistent with established epidemiologic evidence on hearing thresholds in the general population. In the single-outcome models, increasing age was associated with higher thresholds across multiple outcomes, and men had higher thresholds than women for HF-PTA, PTA at 4 kHz, and PTA at 8 kHz. Moreover, repeated-measures ANCOVA showed statistically significant Frequency × age and Frequency × sex interactions, indicating that the effects of age and sex on hearing thresholds are not uniform across frequencies. Previous population-based studies have shown that pure-tone thresholds worsen with age and that men generally have poorer high-frequency hearing than women, particularly at 4 kHz and 8 kHz (11, 12). Therefore, the persistence of an independent altitude-stratified effect at 8 kHz after adjustment for age and sex strengthens the credibility of this finding and further highlights the importance of adequately controlling for these fundamental confounders in studies of hearing in high-altitude populations.

Several limitations of this study should be acknowledged. First, this was a single-center cross-sectional study; although associations between residential altitude strata and hearing thresholds were observed, causal inference cannot be established. Second, the study population was restricted to plateau residents aged 50 years or younger undergoing health examination. While this design helped reduce confounding by age-related hearing decline, it may also limit the generalizability of the findings to older adults and to populations in other high-altitude regions. Third, hearing was assessed primarily by pure-tone audiometry, without additional OAE or ABR measurements, making it difficult to further distinguish the relative contributions of peripheral cochlear dysfunction and central auditory pathway involvement. Fourth, this study used prespecified mean bilateral thresholds as the main audiological outcomes. Marked interaural asymmetry was not used as an *a priori* exclusion criterion or primary analytic stratifier. Although participants with known otologic disease, conductive abnormalities, and type B/C tympanograms were excluded, the use of bilateral mean thresholds may have attenuated clinically relevant information related to asymmetric high-frequency hearing loss. In a *post hoc* descriptive check, marked interaural asymmetry at 8 kHz, defined as an interaural threshold difference of ≥20 dB, was identified in 19 of 187 participants and was not concentrated in a single altitude stratum. Excluding these participants did not reverse the direction of the altitude-stratified pattern, but the fully adjusted association was attenuated. Therefore, the association observed at 8 kHz should be interpreted with caution, and interaural asymmetry should be more systematically evaluated in future studies. Fifth, although participants with a definite history of occupational noise exposure were excluded, detailed lifetime data on recreational and environmental noise exposure were not available. Residual confounding from earphone or headphone use, amplified music exposure, transportation-related noise, military or firearm exposure, and intermittent impulse-noise exposure may have contributed to high-frequency threshold differences, particularly at 8 kHz. Therefore, the observed association at 8 kHz should be interpreted as an altitude-stratified association after adjustment for available covariates, rather than definitive evidence of a direct altitude effect. Finally, hematologic indices were not available for all participants, and formal mediation analyses were not performed; therefore, the specific role of erythrocyte-related adaptation in the association between altitude and hearing thresholds remains exploratory. Despite these limitations, the present study still has clinical relevance. In long-term high-altitude residents, altitude-related hearing-threshold differences appear to have a certain frequency-dependent tendency, and 8 kHz may represent a relatively robust early audiological observation window that complements conventional speech-frequency measures. Larger, multicenter, and prospective studies incorporating OAE, ABR, and more comprehensive hypoxia-related and hematologic markers will be valuable for clarifying the mechanisms linking long-term high-altitude residence, hypobaric hypoxic adaptation, and auditory change.

In summary, this study showed that among plateau residents aged 50 years or younger undergoing health examination, pure-tone hearing thresholds differed across long-term residential altitude strata, and these differences were most apparent at higher frequencies in unadjusted analyses. After further adjustment for age, sex, and other clinical factors, the independent altitude-stratified effect was retained mainly at 8 kHz, whereas the between-group differences in HF-PTA, 4 kHz, and low−/mid-frequency PTA were markedly attenuated or no longer statistically significant. Repeated-measures ANCOVA in the fully adjusted sensitivity model did not show a statistically significant Frequency × altitude group interaction, indicating that the present study does not support a clear overall frequency-specific interaction pattern. Therefore, 8 kHz may be a relatively robust audiological observation point for detecting auditory changes associated with long-term high-altitude residence. Further large-scale, multicenter, and prospective studies incorporating OAE, ABR, and more comprehensive hypoxia-related and hematologic markers are needed to better elucidate the mechanisms underlying the association between residential altitude and auditory change.

## Data Availability

The datasets presented in this article are not readily available because the dataset contains individual-level clinical data and is subject to privacy and confidentiality restrictions; therefore, it is not publicly available. Requests to access the datasets should be directed to Yi Wang, wegreatgroup@163.com; Bin Guo, guobin.3a@163.com.
