# Supplementary material for: Association of residential altitude with pure-tone hearing thresholds in plateau residents aged ≤50 years: a cross-sectional study
Source: Front Neurol. 2026 Jun 16;17:1862718. doi: 10.3389/fneur.2026.1862718 (PMC13314425; doi:10.3389/fneur.2026.1862718)
Supplement: Supplementary file 1 [file Table_1.DOCX]

**Supplementary Materials**

*Exploratory Nonlinear Analyses for PTA at 8 kHz*

Supplementary Methods: Restricted cubic spline analysis and model diagnostics

Restricted cubic spline analysis was performed as an exploratory supplementary analysis to visualize the association between residential altitude and PTA at 8 kHz in the fully adjusted model. A 4-knot natural cubic spline was used, with total knots placed at 2.25, 2.85, 3.66, and 4.08 km. The fully adjusted model included age, sex, ethnicity, smoking history, alcohol consumption, hypertension, hyperlipidemia, OSAHS, and duration of residence. Overall and nonlinearity P values were derived from nested F tests. Model diagnostics included inspection of residual-versus-fitted plots and influence diagnostics. No single observation was identified as having excessive influence based on Cook’s distance >1. Because the spline analysis was exploratory and the confidence intervals widened at the upper altitude range, the findings were interpreted cautiously.

**Supplementary Table S1. Exploratory quadratic model of residential altitude and PTA at 8 kHz in the fully adjusted model.**

| **Term** | **β / ΔF** | **SE** | **95% CI** | **P value** |
| --- | --- | --- | --- | --- |
| Centered residential altitude (per 1 km) | 4.50 | 2.37 | -0.18 to 9.18 | 0.059 |
| Centered residential altitude squared | -5.64 | 2.99 | -11.53 to 0.25 | 0.061 |
| Quadratic vs linear-only model | 3.57 | — | — | 0.061 |

Notes: N = 186 complete cases. The fully adjusted model included age, sex, ethnicity, smoking, drinking, hypertension, hyperlipidemia, OSAHS, and residential years. ALT_C denotes centered residential altitude in kilometers; ALT_C2 denotes the squared centered term. The last row reports the nested-model comparison between the quadratic and linear-only models.

**Supplementary Figure S1. Restricted cubic spline for residential altitude and adjusted PTA at 8 kHz in the fully adjusted model.**


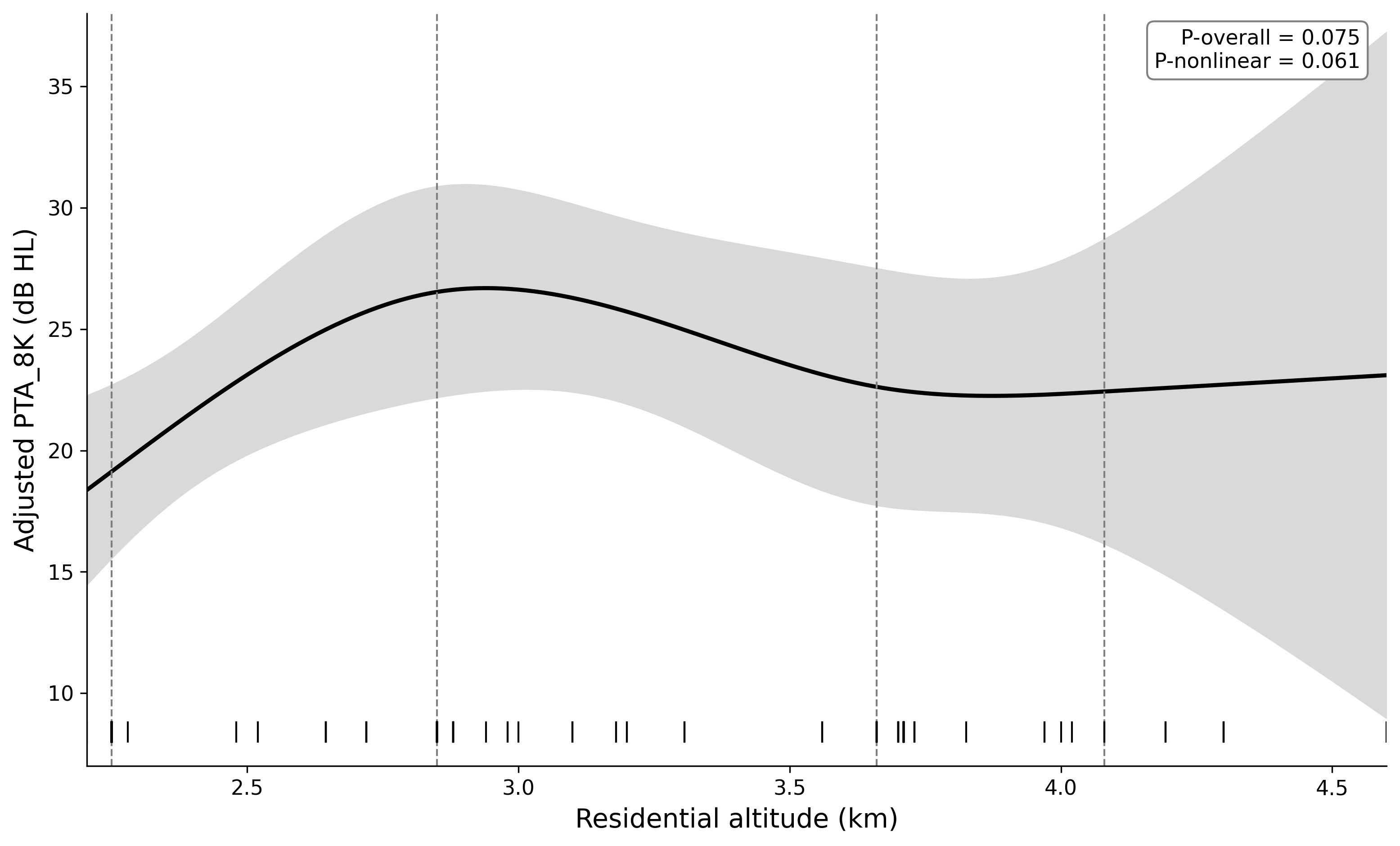


Notes: The solid line shows the marginally adjusted predicted PTA at 8 kHz, and the shaded area indicates the 95% confidence interval. Rug marks denote the observed residential altitude distribution. Total spline knots were placed at 2.25, 2.85, 3.66, and 4.08 km. Overall association P = 0.075 and nonlinearity P = 0.061, both derived from nested F tests. Results suggested a possible non-linear trend, but both the overall association and the test for non-linearity remained exploratory.
