# Supplementary material for: Association of residential altitude with pure-tone hearing thresholds in plateau residents aged ≤50 years: a cross-sectional study
Source: Front Neurol. 2026 Jun 16;17:1862718. doi: 10.3389/fneur.2026.1862718 (PMC13314425; doi:10.3389/fneur.2026.1862718)
Supplement: Supplementary file 2 [file Table_2.DOCX]

**Supplementary Table S2. Fully adjusted repeated-measures ANCOVA examining the interaction between residential altitude group and frequency**

| **Panel** | **Effect / Test** | **Statistic** | **P value** | **Partial η²** |
| --- | --- | --- | --- | --- |
| A | Mauchly’s test of sphericity for Frequency | W = 0.0116; χ² = 762.20; df = 14 | <0.001 | — |
| A | Greenhouse–Geisser epsilon | ε = 0.4137 | — | — |
| B | Frequency × altitude group (Greenhouse–Geisser corrected) | F(4.137, 357.830) = 1.273 | 0.280 | 0.015 |

***Repeated-measures ANCOVA was fitted with PTA at 250*** ***Hz, PTA at 500*** ***Hz, PTA at 1 kHz, PTA at 2 kHz, PTA at 4 kHz, and PTA at 8 kHz as within-subject repeated outcomes. The fully adjusted model included age and residential duration as covariates, and sex, ethnicity, smoking history, alcohol use, hypertension, hyperlipidemia, OSAHS, and residential altitude group as between-subject factors. Because Mauchly’s test indicated violation of sphericity, Greenhouse–Geisser-corrected results are reported.***
