# Supplementary material for: Association of residential altitude with pure-tone hearing thresholds in plateau residents aged ≤50 years: a cross-sectional study
Source: Front Neurol. 2026 Jun 16;17:1862718. doi: 10.3389/fneur.2026.1862718 (PMC13314425; doi:10.3389/fneur.2026.1862718)
Supplement: Supplementary file 3 [file Table_3.DOCX]

Supplementary Table S3. Distribution of residential altitude in the analytic sample

| **Altitude category** | **n** | **Mean ± SD, km** | **Median (Q1, Q3), km** | **Range, km** |
| --- | --- | --- | --- | --- |
| Lower-altitude stratum | 79 | 2.25 ± 0.03 | 2.25 (2.25, 2.25) | 2.21–2.48 |
| Middle-altitude stratum | 51 | 2.89 ± 0.19 | 2.85 (2.85, 2.96) | 2.52–3.31 |
| Higher-altitude stratum | 57 | 3.81 ± 0.23 | 3.71 (3.70, 3.83) | 3.56–4.60 |

Distribution across RCS knot intervals:

| **RCS altitude interval** | **n** |
| --- | --- |
| ≤2.25 km | 77 |
| >2.25 to ≤2.85 km | 31 |
| >2.85 to ≤3.66 km | 34 |
| >3.66 to ≤4.08 km | 37 |
| >4.08 km | 8 |

RCS knot intervals were based on the total spline knots placed at 2.25, 2.85, 3.66, and 4.08 km.
